# Validation of a commercial intraoral auto-segmentation algorithm against expert-annotated clinical data with comparison to a transparent algorithm

**DOI:** 10.3389/froh.2026.1923460

**Published:** 2026-09-03

**Authors:** Rojin Adabdokht, Manuel Lagravere Vich, Thanandon Imaromkul, Kridsada Tosakparalerd, Victor de Miranda Ladewig, Wenji Cai, Chitian Ji, Hollis Lai

**Affiliations:** 1 Mike Petryk School of Dentistry, Faculty of Medicine & Dentistry, University of Alberta, Edmonton, Alberta, Canada; 2 Tomorrow Smile Digital Healthcare LTD, Vancouver, Canada

**Keywords:** deep learning, artificial intelligence, digital dentistry, image segmentation, interpretable AI, intraoral scan (IOS)

## Abstract

**Introduction:**

Artificial intelligence (AI)-based tooth segmentation has the potential to improve the efficiency and consistency of digital clinical workflows; however, the generalizability of commercially available algorithms on clinical datasets and their interpretability remain limited.

**Methods:**

This study proposed a two-level evaluation method. It evaluated the performance of a black-box commercial algorithm using an expert-annotated clinical reference dataset of 126 intraoral scans representing diverse clinical presentations, including severe crowding, spacing, missing teeth, and dental restorations. Two specialists independently generated reference annotations. In the next level, a transparent two-stage segmentation method combining YOLOv8-based tooth localization and numbering with a 3D U-Net segmentation model was developed using 80 scans from an open-source dataset and evaluated alongside the commercial algorithm. Performance was evaluated using the Dice Similarity Coefficient (DSC) and tooth classification accuracy. Tooth detection and numbering were assessed descriptively using confusion matrices and accuracy. End-to-end segmentation performance was compared at the scan level using paired parametric or nonparametric tests, depending on the distribution of paired differences. Regional performance differences between anterior and posterior regions and between maxillary and mandibular jaws were assessed using independent parametric or nonparametric tests, as appropriate.

**Results:**

The commercial algorithm achieved significantly higher segmentation performance than the transparent model, with a median DSC of 0.90 (IQR 0.86–0.95) versus 0.76 (IQR 0.66–0.84), respectively (*p* < 0.001). In contrast, the transparent model showed tooth-numbering accuracy of 0.92 versus 0.83. No significant differences in DSC were observed between anterior and posterior regions for either algorithm. Maxillary and mandibular performance did not differ significantly for the commercial algorithm, whereas the transparent model performed significantly better in the mandible.

**Discussion:**

The commercial algorithm demonstrated strong segmentation performance on an independent, expert-annotated clinical dataset. Although the transparent approach achieved lower segmentation accuracy, it offered greater methodological transparency and insight into decision-making.

## 1 Introduction

Intraoral scans (IOS), have become essential components of dental practice, particularly in orthodontics, prosthodontics and implant dentistry. They enable efficient storage and sharing of patient records, personalized treatment planning, and enhance dentist-patient communication, which contributes to workflow optimization of dental offices (1, 2).

Digital models facilitate treatment processes and visualization of expected outcomes, improving patient understanding and engagement. It is noteworthy that the success of these depends on the accurate teeth segmentation, which is necessary for effective treatment planning. However, manual tooth segmentation is time-consuming, labour-intensive, and susceptible to error (1, 3, 4). Artificial intelligence (AI)-driven tools, such as automated tooth segmentation, significantly reduce processing time and costs, resulting in a more efficient workflow and a less physically demanding practice (2, 3). However, caution is warranted when adopting these technologies for health data.

Technical validation of AI-driven algorithms is particularly critical in healthcare contexts, including dentistry. Recent studies have highlighted growing concerns regarding the generalizability of AI models when applied to real-world clinical settings. Algorithms that perform well during training and internal validation may exhibit reduced accuracy or reliability when evaluated on external clinical datasets. Therefore, external validation is essential to assess the robustness, reproducibility, and clinical applicability of AI algorithms across diverse patient populations and practice environments (5, 6). Results from validation tests can help users evaluate the AI algorithm's real-world performance (6).

Furthermore, the transparency of an algorithm is critically important in medical AI applications. In clinical environments, the reasoning and decision-making processes need to be understandable to gain the trust of both clinicians and patients, thereby facilitating the adoption of new technologies in healthcare by fostering trust among users (7, 8). Such transparency can be enhanced by model architectures that allow greater visibility into their design, processing steps, and decision-making mechanisms. Transparent testing approaches enable users to examine and validate the internal logic and structure of a program. In contrast to algorithm with undisclosed development details, which evaluates software functionality without access to or knowledge of the underlying decision-making processes, transparent testing assumes that the internal modules of the program are well defined and subject to verification. This approach allows users to clearly understand and assess the sequence of steps through which inputs are transformed into outputs (9).

Although several studies have explored automated tooth segmentation through AI-assisted algorithms, demonstrating high precision in tooth numbering and segmentation, the external validity of most of the available systems is unclear (1–4, 10, 11).

For commercially available AI systems, obtaining expert feedback is essential for algorithm performance, applicability, and adaptability improvement. Particularly in clinical image processing, where accuracy and reliability are critical (6).

Therefore, the objective of this study was to conduct a technical validation of a commercially available intraoral scanner segmentation algorithm (Tomorrow Smile Digital Healthcare LTD.) through developing and comparing a transparent methodology, using an expert-developed clinical dataset.

## 2 Materials and methods

### 2.1 Ethics approval

This study was approved by the University of Alberta's Research Ethics Boards (Pro00144987).

### 2.2 Datasets

Two independent datasets were used in the current study: one for training the transparent algorithm and a separate reference dataset for the clinical validation of both algorithms. The use of independent data sources for training and validation ensured no overlap between the development and external validation datasets. Table 1 presents the key characteristics of the two datasets.

**Table 1:** Summary of the datasets. Dataset summary, including scan types, number of scans, purpose, scanner used, and annotation details.

Dataset Type	Scan Type(s)	Number of Scans	Purpose	Scanner	Annotators
Open-access	Oral Scans *(n* *=* *43)* Dental Models *(n* *=* *37)*	80 Mandible *(n* *=* *48)* Maxilla *(n* *=* *32)*	Training and internal validation	Unknown	Unknown annotators
Clinical Data	Oral scans	126 Mandible *(n* *=* *65)* Maxilla *(n* *=* *61)*	External validation	iTero	Two experts (an Orthodontist and a periodontist)

#### 2.2.1 Inclusion and exclusion criteria

Scans from patients with permanent dentition and without orthodontic retainers were included. To enhance external validity and reflect real-world clinical conditions, the validation set included a variety of cases, from normal occlusion to severe crowding or spacing. All included patients exhibited fully erupted first molars (3).

#### 2.2.2 Training dataset

For the training and internal validation of the transparent algorithm, an open-access dataset was used [16]. This dataset comprised intraoral scans and dental model scans in the form of Object (.obj) files. It also included crown segmentations and the corresponding tooth numbers for each tooth in JSON format, with per-vertex tooth labels and tooth instance identifiers. A total of 80 intraoral and dental model scans were used for training. The data and corresponding segmentations were converted into standard tessellation language (.stl) files to facilitate further analysis. STL files are three-dimensional (3D) surface representations of objects. They consist of a mesh of small triangles (called facets) that together define the shape of the object's surface. Each triangle is described by the coordinates of its three vertices and a normal vector that indicates the outward direction of the surface (12).

#### 2.2.3 Clinical validation dataset

For external technical validation, a reference dataset comprising 126 expert-annotated intraoral scans was developed. No dental model scans were included in the validation dataset. Scans were retrospectively identified from records acquired between 2022 and 2025 using iTero Element 5D scanners during routine clinical care at the University of Alberta Orthodontics Clinic. A consecutive eligibility-based sampling approach was used. Beginning with the most recently acquired scans in the iTero repository, records were reviewed in reverse chronological order and assessed against the predefined inclusion and exclusion criteria. All eligible scans encountered during this process were included until the required sample size was reached; therefore, scans were not randomly selected.

Scans had been obtained by different operators as part of routine clinical procedures, thereby reflecting variability in clinical acquisition. The resulting dataset included a broad range of clinical presentations, including moderate to severe malocclusion characterized by crowding or spacing, dental restorations, missing teeth, and crowns. All scans were available in STL file format and were downloaded, anonymized, and assigned study-specific codes before annotation and analysis. The manufacturer confirmed that the validation scans and corresponding patients were independent of the datasets used for the development or previous testing of the commercial algorithm.

The target sample size was determined pragmatically based on the availability of eligible scans during the study period and the feasibility of expert annotation, with the aim of obtaining a sufficiently large and clinically heterogeneous dataset for scan-level external validation.

##### 2.2.3.1 Annotation

Two specialists [one orthodontist (V.ML) and one periodontist (W.C)] independently labelled scans using the MeshLabeler annotation tool [4]. To ensure annotation consistency, an instruction manual was created, and both annotators received training through a live session and a recorded demonstration. The annotators were instructed to label each facet with only one tooth. The annotators labelled the first 10 scans as part of a pilot phase for calculating inter-annotator agreement (IAA). Inter-annotator agreement (IAA) was assessed at the individual-tooth level using the Dice Similarity Coefficient (DSC). A DSC <0.90 was predefined as the threshold for joint review and reannotation. After the initial calibration, the annotators completed a second calibration session before independently annotating the remaining scans. Tooth-level DSC was then calculated for all corresponding annotations. As agreement remained high across all tooth segments, no further consensus review was required. For the final reference dataset, one of the two expert annotations was randomly selected for each tooth and used for subsequent algorithm evaluation.

### 2.3 Model description

#### 2.3.1 Commercial algorithm (tomorrow smile digital healthcare LTD.)

This algorithm is a proprietary system developed by Tomorrow Smile Digital Healthcare LTD., with limited publicly available information regarding its underlying architecture, training data, and development process. The commercial algorithm evaluated in this study was version 1.0.7, released on June 24, 2025, and deployed using the Docker image teeth-segmentor:1.0.7.

The algorithm was applied to the validation set using the manufacturer-provided default configuration. The input data were processed in accordance with the algorithm's standard usage protocols, and output labels and segmentations were extracted for downstream comparison against expert annotations.

##### 2.3.1.1 Post-processing

The commercial algorithm produced surface meshes in STL format, these meshes were voxelized and converted to 128 × 128 × 128 NumPy masks using the same voxelization procedure and spatial resolution applied during preprocessing of the transparent method.

#### 2.3.2 Transparent algorithm

To provide users with insight into the decision-making process of the segmentation algorithm, a transparent algorithm was developed and trained.

##### 2.3.2.1 Tooth detection and numbering

###### 2.3.2.1.1 Preprocessing

To reduce reliance on large-scale annotated training datasets and minimize irrelevant information during model training, we designed a two-step approach that combines noise reduction and sequential attention to individual teeth. This design directs the algorithm's focus to specific regions of interest (ROIs), improving training efficiency and reducing data annotation burden.

This approach was also conceptually grounded in the global-focal model of radiographic assessment. According to this model, expert radiologists first form a holistic impression of the image to identify regions likely to contain abnormalities and then allocate their cognitive resources to these regions for detailed analysis and decision-making. Our model emulates this strategy by first identifying probable areas of interest and then refining its analysis at the tooth level (13, 14).

**Figure 1:**
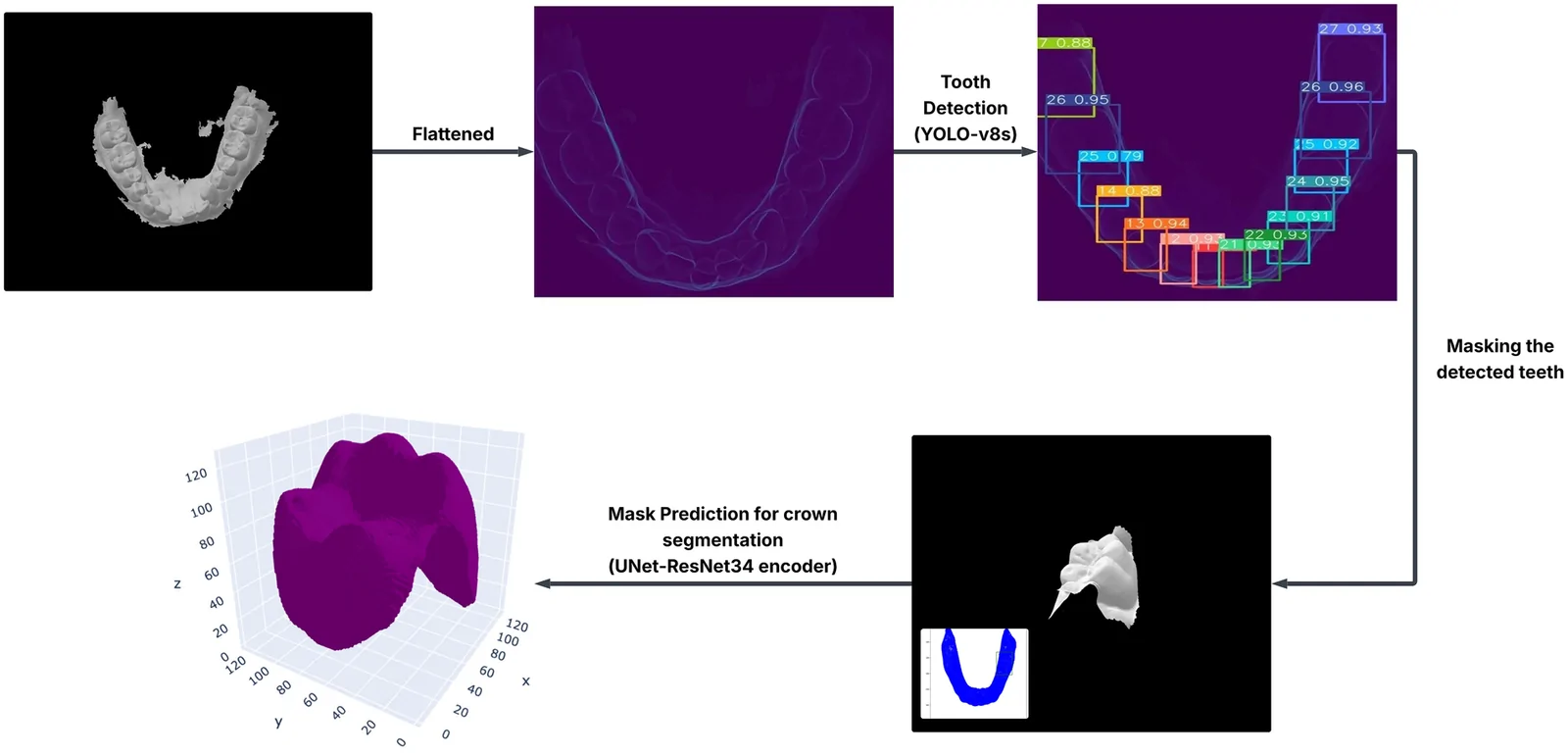
Overview of the system workflow. (1) STL flattening to 2D; (2) tooth detection and labelling using YOLO; (3) STL masking based on detected teeth; and (4) 3D tooth segmentation using a 3D U-Net architecture.

The transparent approach was designed using two consecutive pretrained Convolutional Neural Networks (YOLO-v8 and 3D U-Net), as illustrated in Figure 1 (15, 16).

The scans from the open-access dataset were loaded using the Trimesh library, and their initial dimensions were calculated (17). Meshes were then voxelized using either a fixed voxel size of 0.08 or dividing the smallest mesh dimension by 500, whichever was larger. This strategy preserved mesh detail while maintaining algorithm efficiency, as smaller voxel sizes increase computational demand.

The 3D voxel arrays were then flattened along the z-axis to produce 2D representations of each mesh. These were saved as high-resolution JPEG images (500 DPI). All 2D images, whether mandibular or maxillary, were uniformly oriented with the dental arch facing downward. These images were annotated using the Roboflow annotation tool (18). A calibrated general dentist (R.A) labelled each tooth using polygonal annotations under standardized lighting and monitor conditions. Twelve distinct tooth labels were used. When the dentition was oriented downward, teeth located to the left of the midline were assigned labels in the 1x series, and teeth located to the right of the midline were assigned labels in the 2x series, irrespective of whether they were maxillary or mandibular. The value of x corresponded to tooth type, numbered sequentially from 1 (central incisor) to 6 (first molar) on each side.

A total of 80 annotated images were used to train a YOLOv8 object detection model. For each annotated polygon, the corresponding bounding box was generated and used as the object detection label. After that, the dataset was randomly split into training (80%, *n* = 64), validation (12% *n* = 10), and testing (8%, *n* = 6) sets. To improve the diversity and representativeness of the training data, data augmentation techniques were applied, including random rotations (±14°), brightness adjustments (±12% to ±15%), and exposure modifications (±5%). After augmentation, the training set expanded to 195 images. All images were resized to 640 × 640 pixels.

###### 2.3.2.1.2 Training

A pretrained YOLOv8-small model was fine-tuned for 60 epochs using a batch size of 16, a learning rate of 0.01, the Adam optimizer, and an early stopping patience of five epochs. Mosaic augmentation was disabled during the final 10 epochs of training. No layers were frozen, no dropout was applied, and an intersection over union (IoU) threshold of 0.7 was used. The model weights corresponding to the highest validation performance were saved.

###### 2.3.2.1.3 Post-processing

Predicted boxes were exported as a CSV file containing the image name, predicted tooth number (class), and the coordinates of the predicted bounding boxes (Xmin, Ymin, Xmax, Ymax). The position of each predicted bounding box relative to the dental midline was evaluated to verify that the model correctly assigned left and right tooth labels. The proportion of the bounding box area located on each side of the midline was used rather than the tooth center, thereby reducing the risk of incorrect labeling of the central incisors in cases with dental yaw or midline rotation. The exported coordinates were then used to mask and crop the original input mesh files. To accommodate the height of contour and tilted teeth, bounding boxes were expanded by 5 pixels in all directions.

Vertices within each bounding box were isolated, and their indices were used to extract corresponding mesh facets. Each tooth was then cropped and saved as an individual STL file labelled with the appropriate tooth number.

##### 2.3.2.2 Tooth segmentation

###### 2.3.2.2.1 Preprocessing

The tooth segments extracted using the predicted bounding boxes, together with the corresponding crown segmentations from the open-access dataset, were voxelized using a uniform voxel size of 0.1 mm. Each voxelized segment was resized to a resolution of 128 × 128 × 128 voxels using nearest-neighbor interpolation to preserve the integrity of the binary masks while optimizing computational efficiency. The resulting voxelized tooth volumes and their corresponding crown segmentations were stored in Hierarchical Data Format version 5 (HDF5).

###### 2.3.2.2.2 Training

A 3D U-Net model with a ResNet-34 encoder (19) was trained using 921 tooth segments. To prevent data leakage between scans, the dataset was first partitioned at the scan level and subsequently divided into training (80%; *n* = 737) and validation (20%; *n* = 184) sets. The model was trained for 30 epochs with a batch size of 4 using the Adam optimizer, a learning rate of 0.001, and a cross-entropy loss function. Early stopping with a patience of five epochs was applied based on the validation loss. The model generated a binary segmentation mask for each tooth segment. Segmentation performance was evaluated using the DSC, calculated with a tolerance of one voxel to account for interpolation errors.

### 2.4 Technical external validation

Both algorithms were evaluated using the clinical validation dataset. To ensure the security and privacy of clinical data, the commercial algorithm was executed locally on a secure host computer at the University of Alberta. The developers provided a Dockerfile that enabled the algorithm to run in a controlled local environment without transferring patient data outside the institution.

The performance of the algorithms was evaluated for tooth detection, tooth classification, and tooth segmentation. Detection and classification performance were assessed simultaneously based on the predicted tooth bounding boxes and assigned tooth labels. Overall accuracy was calculated as the proportion of correctly detected and classified teeth relative to the reference annotations, and confusion matrices were generated to identify patterns of misclassification among tooth types. Tooth segmentation performance was evaluated using the DSC, which quantifies the spatial overlap between the predicted segmentation and the reference annotation.

The reference annotations were converted into voxel grids using the same preprocessing process applied to the transparent model. All representations were generated in the same coordinate space; therefore, no additional image registration or alignment transformation was applied. DSC was subsequently calculated between the resulting voxel masks. The one-voxel tolerance used in the evaluation was applied equally to both the transparent and commercial algorithm outputs.

Predicted teeth were matched to the corresponding reference teeth using an automated matching algorithm. The evaluation was performed as an end-to-end assessment of the system; therefore, missed detections, incorrect tooth labels, merged teeth, split segmentations, partially scanned teeth, and other unsuccessful predictions were retained as failures rather than excluded from the analysis and missed detections received DSC of 0. Accordingly, the reported DSC reflects overall end-to-end system performance, incorporating both detection/identification failures and segmentation accuracy, rather than segmentation performance conditional on successful detection.

Because both algorithms were evaluated on the same clinical scans, the comparison was treated as paired. To account for clustering of multiple teeth within each scan, tooth-level DSC values were aggregated by calculating the mean DSC for each scan for each algorithm. The scan was therefore used as the unit of analysis. Normality of the paired DSC differences between the two algorithms was assessed using the Shapiro–Wilk test. When the paired differences did not significantly deviate from a normal distribution, comparisons were performed using a paired-samples t-test. When the paired differences were not normally distributed, the Wilcoxon signed-rank test was used. Statistical significance was set at *p* < 0.05.

## 3 Results

### 3.1 Dataset

From the open-access dataset, 80 were selected for algorithm development. These scans included 48 mandibular scans and 32 maxillary scans. Additionally, 43 were oral scans, while 37 were scans of dental models.

The validation set included 126 scans comprising 1,464 teeth from 88 unique patients. Of these, 65 were mandibular scans and 61 were maxillary scans. Thirty-eight patients contributed both maxillary and mandibular scans, while the remaining 50 patients contributed a single-jaw scan. Among the 126 scans, 60 exhibited moderate to severe malocclusion, including dental crowding or spacing, whereas the remaining 66 demonstrated normal occlusal relationships or mild malocclusion (<3 mm). The dataset also included scans with missing teeth and dental restorations, including fillings and crowns, further contributing to the clinical heterogeneity of the validation cohort.

#### 3.1.1 Inter-annotator agreement (IAA)

During the initial calibration phase, tooth-level DSC values were ≥0.90 for all annotations. High inter-annotator agreement was maintained throughout the remaining dataset, with a mean DSC of 0.97 ± 0.02 and values ranging from 0.91 to 1.00. As all tooth-level DSC values remained above the predefined threshold of 0.90, no additional consensus review was required.

### 3.2 Tooth detection and numbering

#### 3.2.1 Transparent training

The tooth detection results showed strong learning performance across all evaluation metrics in localizing the teeth area. The model achieved high overall precision and recall for tooth detection, with a mean average precision at the IoU threshold of 0.5 (mAP@0.5) of 0.98 across all tooth classes. Class-specific mAP scores ranged from 0.93 to 0.99.

#### 3.2.2 Clinical validation

**Figure 2:**
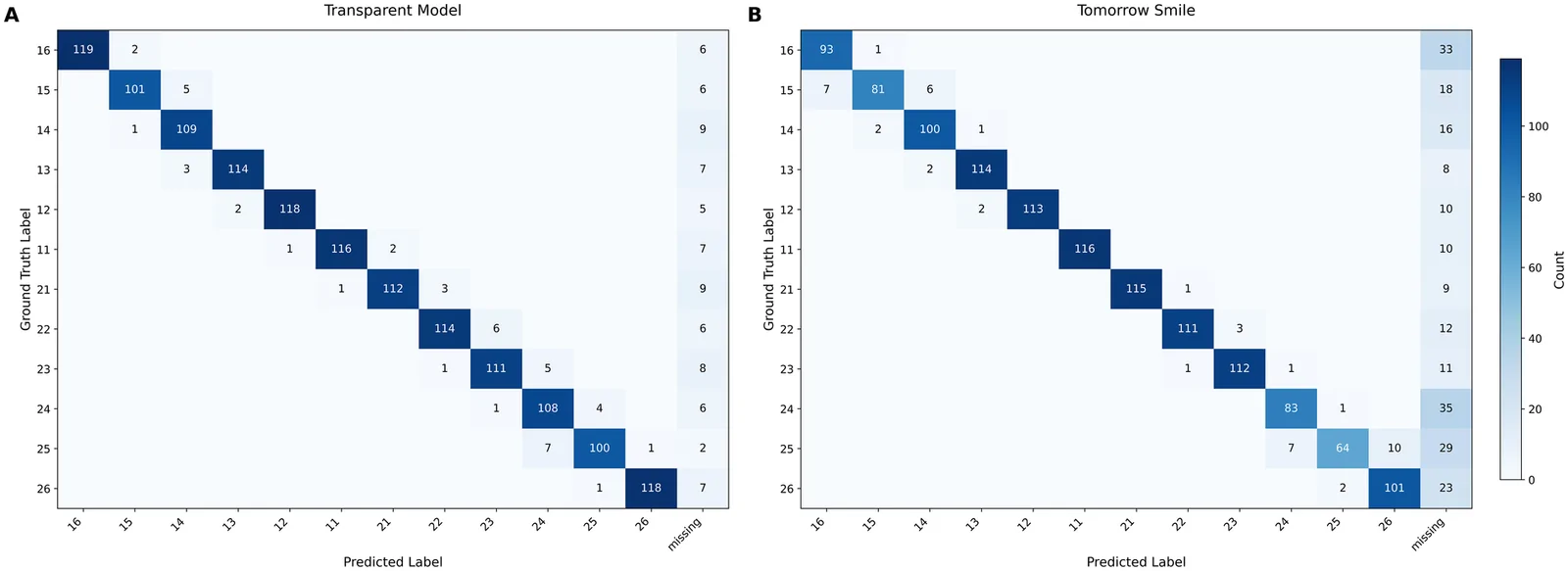
Confusion matrices comparing tooth detection and numbering. **(A)** The transparent approach and **(B)** the Tomorrow Smile algorithm. Diagonal cells indicate correct predictions, off-diagonal cells indicate misclassifications, and the “missing” column represents missed detections.

The Tomorrow Smile algorithm achieved a mean tooth classification accuracy of 0.83 (SD = 0.38), whereas the transparent approach demonstrated a higher overall accuracy of 0.92 (SD = 0.28). Figure 2 illustrates the comparative external validation of classification performance of the two algorithms.

### 3.3 Tooth segmentation

#### 3.3.1 Transparent training

Training and validation losses steadily decreased throughout training. The model started with a training loss of 0.31 and a validation loss of 0.65. By the final epoch, the training and validation losses decreased to 0.15 and 0.17, respectively, with a relatively small difference between them.

#### 3.3.2 Clinical validation

On external validation, the transparent model demonstrated moderate segmentation performance. Segmentation performance was significantly higher for the mandibular dentition than for the maxillary dentition. However, no statistically significant difference was observed between the segmentation performance of anterior and posterior teeth.

The Tomorrow Smile algorithm demonstrated consistently high segmentation performance across all evaluated regions and arches. No statistically significant differences in segmentation performance were observed between the maxillary and mandibular arches or between anterior and posterior teeth.

**Figure 3:**
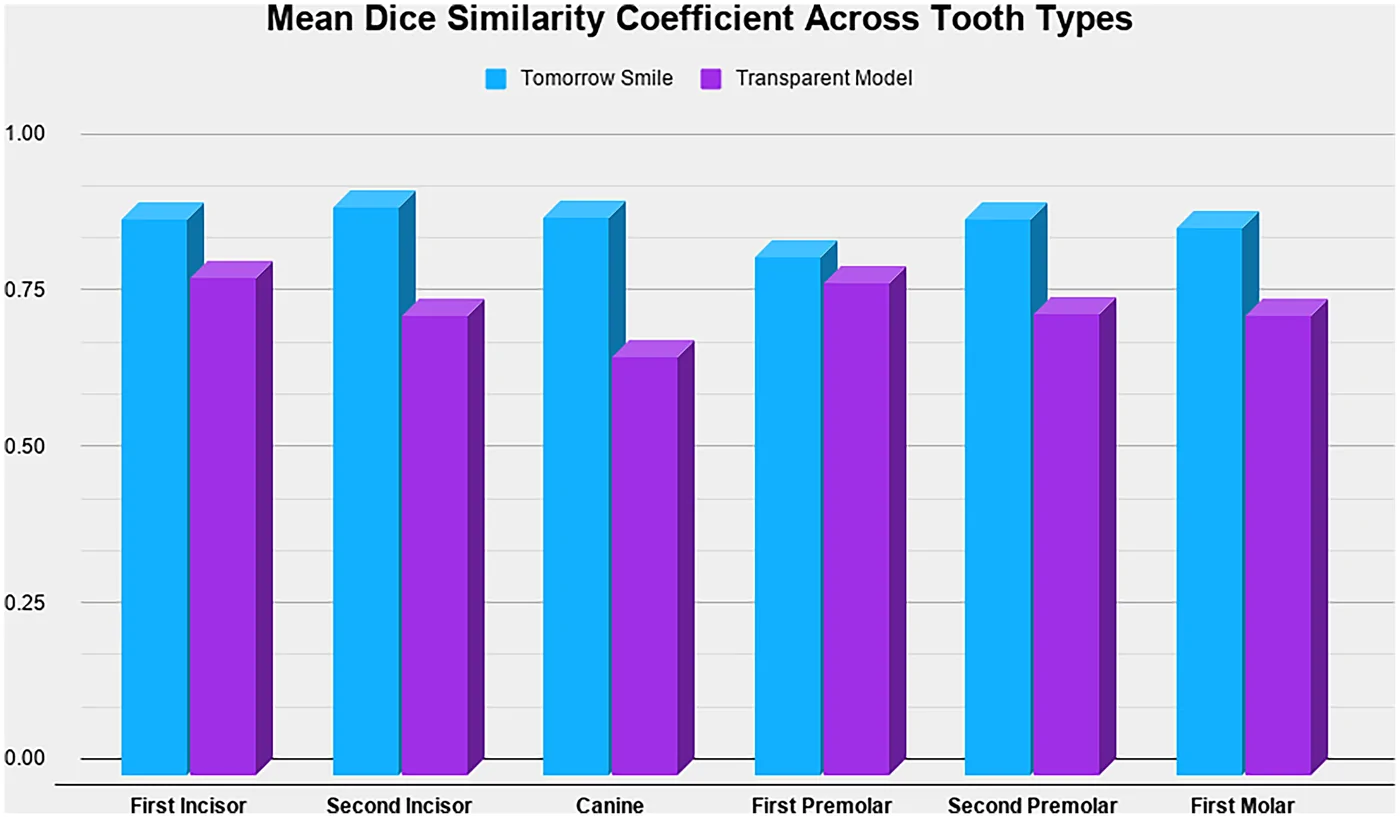
Mean dice similarity coefficient (DSC) for each tooth type. Comparing the Tomorrow Smile and transparent approaches. Higher DSC values indicate greater agreement between predicted and reference segmentations.

**Figure 4:**
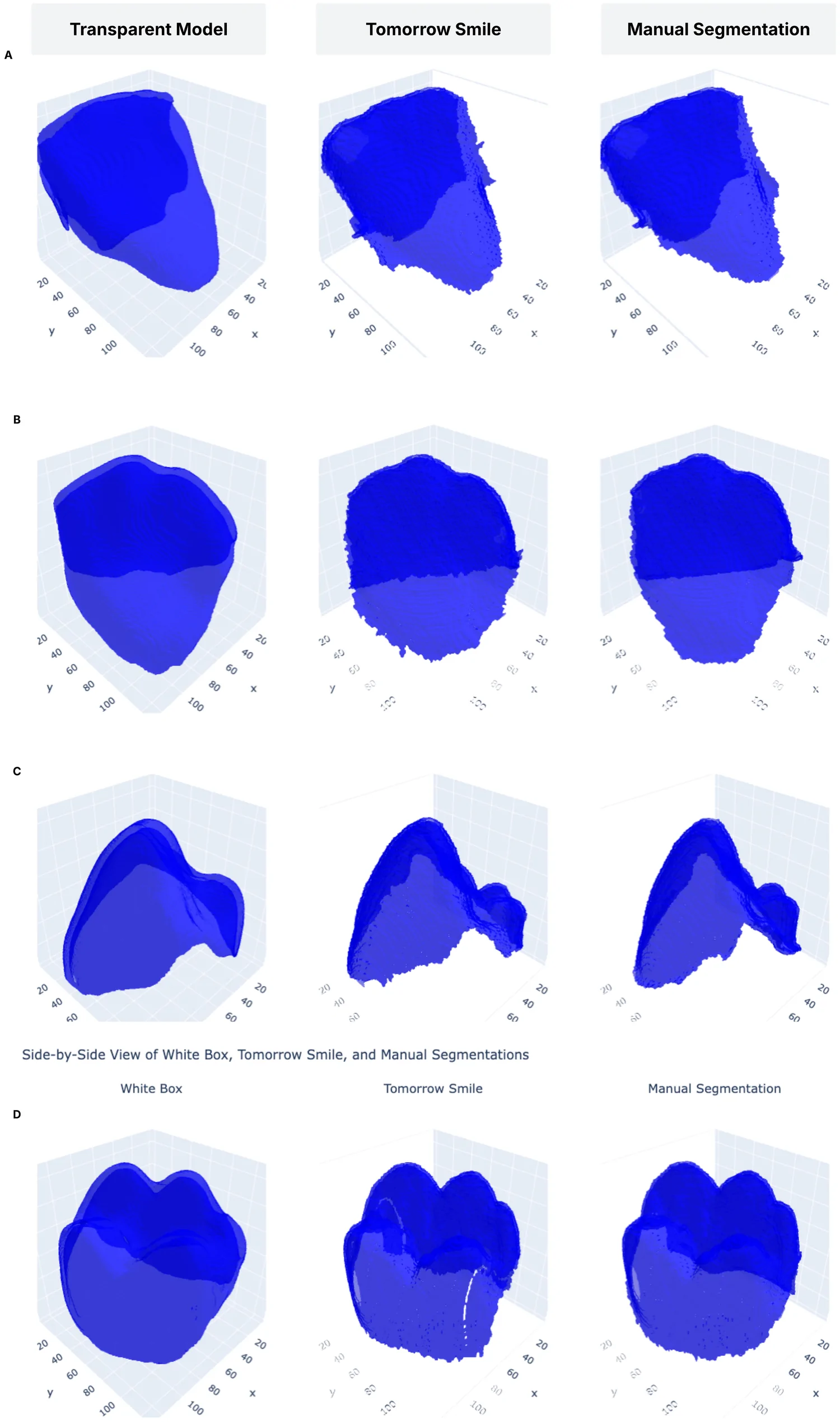
Segmentation comparison. Comparative visualization of mandibular segmentation outputs generated by transparent model, Tomorrow Smile, and manual segmentation. **(A)** Central incisor in lingual view. **(B)** Canine in lingual view. **(C)** First premolar in mesial view. **(D)** First molar in distolingual view. Segmentation models are displayed with 50% transparency to facilitate spatial comparison; darker shading denotes posterior regions.

Regional and arch-based analyses are summarized in Table 2, and performance across individual tooth types is illustrated in Figure 3. Figure 4 further illustrates segmentation examples from transparent model, Tomorrow Smile, and manual segmentation.

**Table 2:** Model performance on the external test dataset.

Subgroup	Transparent Model Median DSC (IQR 1-3)	Tomorrow Smile Median DSC (IQR 1-3)	Test	*p*-value
All teeth	0.76 (0.66–0.84)	0.90 (0.86–0.95)	Student's t-test	**<0**.**001**
Posterior teeth	0.77 (0.67–0.88)	0.91 (0.83–0.97)	Student's t-test	**<0**.**001**
Anterior teeth	0.75 (0.63–0.86)	0.92 (0.85–0.99)	Wilcoxon signed-rank test	**<0**.**001**
Anterior vs. Posterior	*p* = 0.28	*p* = 0.13	Mann–Whitney u test	**-**
Mandibular arch	0.82 (0.77–0.86)	0.90 (0.87–0.95)	Wilcoxon signed-rank test	**<0**.**001**
Maxillary arch	0.67 (0.60–0.75)	0.92 (0.83–0.95)	Student's t-test	**<0**.**001**
Maxillary vs. Mandibular	**<0.001**	*p* = 0.62	Mann–Whitney U test	-

Median Dice similarity coefficient (DSC) values and interquartile ranges (IQR) for the transparent model and tomorrow smile algorithm, overall and stratified by tooth region and dental arch. Statistical comparisons were performed at the scan level to account for clustering of teeth within scans.

Bold values indicate statistically significant differences (*p* < 0.05).

To compare the end-to-end segmentation performance of the two models, the distribution of paired scan-level DSC differences was first assessed using the Shapiro–Wilk test and did not significantly deviate from normality (*p* = 0.124). Accordingly, a paired-samples t-test was used to compare DSC values between the two algorithms. Overall, the commercial algorithm achieved significantly higher DSC values than the transparent algorithm, t (125) = 10.59, *p* < 0.001. Similarly, subgroup comparisons by dental arch and tooth region demonstrated significantly higher segmentation performance for the commercial algorithm across all comparisons (*p* < 0.001).

## 4 Discussion

With the rise of AI-driven software as computer-assisted decision-making tools, the external validation and reliability assessment of these algorithms have become imperative (5). Limited generalizability is one of the main reasons AI-driven algorithms have not been adopted in clinical workflows. External validation of commercialized algorithms can help clinicians make informed decisions on whether and how to employ them in their practice.

Validation of AI systems can be conducted using several methodological approaches, depending on the intended clinical application of the model. Common strategies include model-centered validation and expert opinion-based benchmarking. Model-centered validation is typically performed during the initial evaluation phase to quantify algorithmic performance using predefined metrics. In contrast, expert opinion-based validation relies on assessments from domain experts, whose judgments serve as a reference standard against which the model's outputs are compared.

Additionally, beyond external validation, the explainability and interpretability of AI systems significantly affect their market readiness through gaining end users’ trust (8). This is especially important in clinical environments, where understanding the decision-making process is essential to rule out incorrect diagnoses and predictions (20). It is noteworthy that the applied AI in dentistry is increasingly dominated by proprietary vendor-based solutions, where limited transparency regarding algorithm design and training processes creates challenges for independent validation and scientific reproducibility (21, 22). The current study proposes a novel validation framework that incorporates a transparent comparator algorithm to systematically characterize the performance, strengths, and limitations of a proprietary commercial system. In a subsequent phase, the technical performance of the tooth segmentation algorithms was externally validated using an expert-annotated clinical dataset. This multi-stage strategy enabled both model-level comparison using a transparent comparator algorithm and expert-driven clinical assessment of the commercial system. Prior evaluations of commercially available auto-segmentation software have offered useful performance assessments, yet the applied evaluation metrics largely overlooked spatial similarity and geometric correspondence of the segmented outputs. These studies primarily focused on tooth identification and mesiodistal width estimation, relying on manual qualitative scoring approaches that lack standardized quantitative measures for assessing three-dimensional overlap and morphological agreement (3).

Accurate validation of medical imaging products requires evaluation metrics that are consistent with the technical and representational characteristics of the task, imaging modality and data format. A 2022 study that presented a guideline for medical image segmentation found that F-measure-based metrics such as Dice Similarity Coefficient (DSC) and Intersection-over-Union (IoU) are the most appropriate metrics for segmentation evaluation (23). Moreover, to ensure methodological generalizability, intraoral scans were standardized to the Standard Tessellation Language (STL) format, which is universally supported across intraoral scanners, unlike alternative mesh formats (such as PLY) that are device-dependent. This format provides a spatial representation of the dentition by approximating its surface with a mesh of small triangles (called facets). Each STL file contains the 3D coordinates (x, y, z) of the vertices of these triangles but does not include any information about the color or texture of the object.

In the current study, to develop the transparent system, we employed a multi-step approach for noise reduction, aiming to limit the ROI size and avoid multiple ROIs in a single segmentation task. This decision was made to make the pipeline more task-oriented. By noise reduction, the algorithm was able to converge without using a large labeled training data. This method was adopted to prevent evaluation challenges associated with simultaneously including multiple regions of interest within a single input data instance (23).

The results showed that both algorithms demonstrated promising performance in tooth segmentation, with DSC values more than 0.70 (23). This result is comparable with previous studies that applied deep learning algorithms for tooth segmentation. On this note, the previous transparent approaches introduced for tooth segmentation also showed high segmentation performance (11). However, the drawback of these segmentation approaches is their requirement of a large number of labelled training data. Training of such algorithms, although results in high-accuracy models, requires extensive time and financial resources for data processing and model development (24). In contrast, the sequential approach used in this study was trained on a relatively small dataset and employed a multi-stage strategy to progressively localize and refine regions of interest (ROIs), thereby reducing the complexity of the segmentation task.

As results suggest, the transparent model achieved a higher DSC when validated internally, on a subset of the data from which the training set was pooled. Similarly, Wang et al. introduced an algorithm with multistep U-Net segmentation approach. According to their report, their voxelized segmentation approach achieved more than 0.95 in DSC for dentitions without any missed tooth (1). Although the results show high performance, the black-box nature of this algorithm brings up several questions. The unreported voxel size, voxelization technique, decoder used for 3D U-Net, how the data was split, and unclear annotator calibration process makes it difficult to interpret the reported results, as all of these factors can affect the validity and interpretation of the results (25).

In external validation, the DSC for the transparent algorithm dropped to 0.74. One possible explanation is that the algorithm's training set included scans from dental models, whereas the clinical test set included only intraoral scans. This heterogeneity between the two datasets could contribute to a decrease in the algorithm's segmentation performance. This is also aligned with the findings from previous studies on how the difference between the training and testing data can affect the model performance (26). Furthermore, the superior performance of the algorithm on mandibular dentition may be attributed to an imbalance in the training dataset, with a disproportionate representation of lower versus upper dentition samples.

When directly compared, the commercially available algorithm demonstrated significantly higher segmentation performance than the transparent algorithm on the evaluated dataset. These findings indicate that, under the conditions assessed in this study, the commercial algorithm achieved greater agreement with the reference manual segmentations. However, this comparison was limited to segmentation performance as measured on the present dataset and does not account for other factors such as availability, usability, processing time, manual correction requirements, workflow integration, or clinical outcomes. Therefore, the findings should be interpreted as a technical comparison of segmentation accuracy rather than as evidence of the overall superiority or clinical utility of either approach. Furthermore, the lower DSC observed in the transparent algorithm may be attributed to differences in segmentation methodology. In the transparent workflow, segmentation was performed on a voxelized NumPy array, whereas the Tomorrow Smile algorithm performed segmentation directly on the input mesh, with only the resulting segmentations subsequently converted to NumPy arrays. This methodological difference may have contributed to the greater morphological similarity between the Tomorrow Smile and manual segmentations, as illustrated in Figure 4. Additionally, more assessments in the clinical settings are needed to identify how clinically significant the differences are between the two algorithms and their potential for workflow integration (27).

Although the overall performance of the commercial algorithm was better compared to the transparent approach, in the tooth-wise comparison, the commercial version got the lowest performance score for the first premolar tooth, while the transparent approach got the highest score for this tooth. Closer inspection revealed frequent over-segmentation errors, where the algorithm merged the first and second premolars into a single entity. In the transparent approach, since the localization and classification took place before the segmentation, the algorithm did not face such confusion (28). On the other hand, the transparent model faced more challenges in segmenting canines. This can be explained through the higher variation of the position of this tooth compared to the other ones (many of the canines in the validation set had erupted buccally or lingually) (28).

### 4.1 Limitations and future research

Although the external validation dataset was moderate in size (*n* = 126), it was deliberately curated to capture variability across scanners, operators, and clinical presentations, thereby enhancing its representativeness of real-world orthodontic practice. Nevertheless, larger multi-center studies would be valuable to further confirm generalizability.

Moreover, segmentation performance was assessed primarily using DSC, which provides information on volumetric overlap but does not fully characterize boundary accuracy or geometric agreement. The transparent model is currently undergoing further development to expand its functionality and enable more detailed spatial analyses and quantitative feature measurements. Future evaluations will therefore incorporate more comprehensive surface-based and geometric metrics.

Furthermore, the training dataset and development process of the commercial model are proprietary; therefore, important characteristics such as the number and composition of training scans, the range of clinical presentations and occlusal conditions represented, and the rigor of the training and validation procedures are unknown. In contrast, the transparent model was trained on a relatively small dataset of 80 scans. Consequently, this study should not be interpreted as a direct methodological comparison between the two approaches, as such a comparison would require models trained and evaluated under comparable conditions. Rather, the present study focuses on comparing their performance on the same independent clinical validation dataset.

It is suggested that the clinical application of the commercially available algorithm be tested at a clinical setting through a mixed method study, combining the quantitative results of a clinical trial accuracy study as well as a qualitative assessment of user-friendliness and clinicians’ thoughts, feelings, and attitudes towards the application of this software.

## 5 Conclusion

This study demonstrated that the commercially available algorithm achieved higher segmentation performance than the transparent segmentation approach on a single-centre, retrospectively curated clinical dataset acquired using the iTero scanner family. The transparent model also demonstrated promising segmentation performance, supporting its potential as a technically viable alternative for further investigation. However, the interpretation of DSC values should be considered in the context of the specific segmentation task and clinical application rather than based on a universal threshold for high or acceptable performance. These findings are limited to the evaluated dataset and imaging environment. Broader generalizability across different centres, scanner systems, patient populations, and clinical workflows, as well as the clinical usefulness of both approaches, requires further external validation.

## Data Availability

The data analyzed in this study is subject to the following licenses/restrictions: Data are not publicly available due to privacy and ethical restrictions but are available from the corresponding author upon reasonable request. Requests to access these datasets should be directed to Hollis Lai, hollis1@ualberta.ca.

## References

[1] Wang X Alqahtani KA Van Den Bogaert T Shujaat S Jacobs R Shaheen E. Convolutional neural network for automated tooth segmentation on intraoral scans. BMC Oral Health. (2024) 24(1):804. 10.1186/s12903-024-04582-2 39014389 PMC11250967

[2] van Nistelrooij N Maier E Bronkhorst H Crins L Xi T Loomans BAC. Automated monitoring of tooth wear progression using AI on intraoral scans. J Dent. (2024) 150:105323. 10.1016/j.jdent.2024.105323 39197530

[3] Yacout YM Eid FY Tageldin MA Kassem HE. Evaluation of the accuracy of automated tooth segmentation of intraoral scans using artificial intelligence-based software packages. Am J Orthod Dentofacial Orthop. (2024) 166(3):282–291.e1. 10.1016/j.ajodo.2024.05.015 38904564

[4] Wu T-H Lian C Lee S Pastewait M Piers C Liu J. Two-Stage mesh deep learning for automated tooth segmentation and landmark localization on 3D intraoral scans. IEEE Trans Med Imaging. (2022) 41(11):3158–66. 10.1109/TMI.2022.3180343 35666796 PMC10547011

[5] Myllyaho L Raatikainen M Männistö T Mikkonen T Nurminen JK. Systematic literature review of validation methods for AI systems. J Syst Softw. (2021) 181:111050. 10.1016/j.jss.2021.111050

[6] Mahajan V Venugopal VK Murugavel M Mahajan H. The algorithmic audit: working with vendors to validate radiology-AI algorithms—how we do it. Acad Radiol. (2020) 27(1):132–5. 10.1016/j.acra.2019.09.009 31818381

[7] Rosenbacke R Melhus Å McKee M Stuckler D. How explainable artificial intelligence can increase or decrease Clinicians’ trust in AI applications in health care: systematic review. JMIR AI. (2024) 3:e53207. 10.2196/53207 39476365 PMC11561425

[8] Ali S Abuhmed T El-Sappagh S Muhammad K Alonso-Moral JM Confalonieri R. Explainable artificial intelligence (XAI): what we know and what is left to attain trustworthy artificial intelligence. Inf Fusion. (2023) 99:101805. 10.1016/j.inffus.2023.101805

[9] Haley A Zweben S. Development and application of a white box approach to integration testing. J Syst Softw. (1984) 4(4):309–15. 10.1016/0164-1212(84)90030-X

[10] Jana A Maiti A Metaxas DN. A critical analysis of the limitation of deep learning based 3D dental mesh segmentation methods in segmenting partial scans. In: 2023 45th Annual International Conference of the IEEE Engineering in Medicine & Biology Society (EMBC) (Sydney, Australia: IEEE) (2023), 1–7. 10.1109/EMBC40787.2023.10339972 38082617

[11] Li J Cheng B Niu N Gao G Ying S Shi J. A fine-grained orthodontics segmentation model for 3D intraoral scan data. Comput Biol Med. (2024) 168:107821. 10.1016/j.compbiomed.2023.107821 38064844

[12] Szilvśi-Nagy M Mátyási G. Analysis of STL files. Math Comput Model. (2003) 38(7–9):945–60. 10.1016/S0895-7177(03)90079-3

[13] Drew T Evans K Võ MLH Jacobson FL Wolfe JM. Informatics in radiology: what can you see in a single glance and how might this guide visual search in medical images? RadioGraphics. (2013) 33(1):263–74. 10.1148/rg.331125023 23104971 PMC3545617

[14] Waite S Farooq Z Grigorian A Sistrom C Kolla S Mancuso A. A review of perceptual expertise in radiology-how it develops, how we can test it, and why humans still matter in the era of artificial intelligence. Acad Radiol. (2020) 27(1):26–38. 10.1016/j.acra.2019.08.018 31818384

[15] Jocher G Qiu J Liu M Lyu S Akyon FC Kalfaoglu ME. Ultralytics YOLO26: Unified real-time end-to-end vision models. Frederick: Ultralytics, Inc. (2026). 10.48550/arXiv.2606.03748 (Accessed January 30, 2026).

[16] Çiçek Ö Abdulkadir A Lienkamp SS Brox T Ronneberger O. 3D U-Net: Learning Dense Volumetric Segmentation from Sparse Annotation. arXiv (2016). 10.48550/ARXIV.1606.06650

[17] Dawson-Haggerty M Bacher J Brown-Bivins A Forest C O’Kane J. trimesh: Load and analyze triangular meshes (Version 3.2.0) [Computer software] (2019). Available online at: https://trimesh.org/ (Accessed November 14, 2025)

[18] Dwyer B Nelson J Hansen T Solawetz J. Roboflow (Version 1.0) [Software] 2024. Available online at: https://roboflow.com. Computer vision (Accessed July 27, 2025).

[19] He K Zhang X Ren S Sun J. Deep Residual Learning for Image Recognition. arXiv (2015). 10.48550/ARXIV.1512.03385

[20] Solberg Hjorth S Brem A. How to assess market readiness for an innovative solution: the case of heat recovery technologies for SMEs. Sustainability. (2016) 8(11):1152. 10.3390/su8111152

[21] Mallineni SK Sethi M Punugoti D Kotha SB Alkhayal Z Mubaraki S. Artificial intelligence in dentistry: a descriptive review. Bioengineering. (2024) 11(12):1267. 10.3390/bioengineering11121267 39768085 PMC11673909

[22] Najjar R. Redefining radiology: a review of artificial intelligence integration in medical imaging. Diagnostics. (2023) 13(17):2760. 10.3390/diagnostics13172760 37685300 PMC10487271

[23] Müller D Soto-Rey I Kramer F. Towards a guideline for evaluation metrics in medical image segmentation. BMC Res Notes. (2022) 15(1):210. 10.1186/s13104-022-06096-y 35725483 PMC9208116

[24] Martín-Noguerol T López-Úbeda P Paulano-Godino F Luna A. Manual data labeling, radiology, and artificial intelligence: it is a dirty job, but someone has to do it. Magn Reson Imaging. (2025) 116:110280. 10.1016/j.mri.2024.110280 39557176

[25] Mongan J Moy L Kahn CE. Checklist for artificial intelligence in medical imaging (CLAIM): a guide for authors and reviewers. Radiol Artif Intell. (2020) 2(2):e200029. 10.1148/ryai.2020200029 33937821 PMC8017414

[26] Hou R Lo JY Marks JR Hwang ES Grimm LJ. Classification performance bias between training and test sets in a limited mammography dataset PLoS One. (2024) 19(2):e0282402. 10.1371/journal.pone.0282402 38324545 PMC10849231

[27] Langner D Nachbar M Russo ML Boeke S Gani C Niyazi M. Comparative analysis of open-source against commercial AI-based segmentation models for online adaptive MR-guided radiotherapy. Z Für Med Phys. (2025):S0939388925000777. 10.1016/j.zemedi.2025.04.008 40345918

[28] Huang Y-J Dou Q Wang Z-X Liu L-Z Jin Y Li C-F. 3D RoI-aware U-net for accurate and efficient colorectal tumor segmentation. IEEE Trans Cybern. (2021) 51(11):5397–408. 10.1109/TCYB.2020.2980145 32248143

